# How old are you? Newborn gestational age discriminates neonatal resuscitation practices in the Italian debate

**DOI:** 10.1186/1472-6939-10-19

**Published:** 2009-11-12

**Authors:** Emanuela Turillazzi, Vittorio Fineschi

**Affiliations:** 1 Department of Legal Medicine. University of Foggia, Ospedale Colonnello D'Avanzo, Via degli Aviatori, 1, 71100 Foggia, Italy

## Abstract

**Background:**

Multidisciplinary study groups have produced documents in an attempt to support decisions regarding whether to resuscitate "at risk" newborns or not. Moreover, there has been an increasingly insistent request for juridical regulation of neonatal resuscitation practices as well as for clarification of the role of parents in decisions regarding this kind of assistance. The crux of the matter is whether strict guidelines, reference standards based on the parameter of gestational age and authority rules are necessary.

**Discussion:**

The Italian scenario reflects the current animated debate, illustrating the difficulty intrinsic in rigid guidelines on the subject, especially when gestational age is taken as a reference parameter for the medical decision.

**Summary:**

Concerning the decision to interrupt or not to initiate resuscitation procedures on low gestational age newborns, physicians do not need rigid rules based on inflexible gestational age and birth weight guidelines. Guidance in addressing the difficult and trying issues associated with infants born at the margins of viability with a realistic assessment of the infant's clinical condition must be based on the infant's best interests, with clinicians and parents entering into what has been described as a "partnership of care".

## Background

In recent decades a lively debate has developed concerning the decision to interrupt or not to initiate resuscitation procedures on low gestational age newborns [1-3]. The flourishing of rulings by multidisciplinary study groups is evidence of the importance of this debate, with neonatologists, paediatricians, obstetricians and bioethicists working togheter in the attempt to support decisions regarding whether to resuscitate "at risk" newborns or not [4,5].

At present, in Italy, there has been an increasingly insistent request for juridical regulation of neonatal resuscitation practices as well as for clarification of the role of parents in decisions regarding this kind of assistance. A look at the operative situation shows that adherence to neonatal resuscitation guidelines is low across Italian tertiary centres. The practice of and approach to the resuscitation of ELBW (Extremely Low Birth Weight) infants varies greatly between the centres surveyed, reflecting a paucity of evidence and consequent uncertainty among clinicians [6,7].

In this scenario emerged the so-called "*Carta di Firenze*", compiled by a group of Italian obstetricians and paediatricians, which in addition to the need to ensure that the mother and the newborn are offered adequate assistance, aims to spare them useless, painful and ineffective therapies [8]. The *Carta di Firenze *basically makes reference to the epidemiological data of the EPICure study [9], defining as "of uncertain vitality" infants born between 22 and 25 weeks of gestational age and classifying the therapies administered to the infant during this period as extraordinarily intensive therapies. The fundamental instructions of the chart can be summarized thus:

▪ 22 weeks' gestational age (154-160 days' intrauterine life). Decisions regarding the treatment of the mother must be based on her health conditions. Caesarean section must be performed only when indicated by the mother's clinical conditions; women requesting it for other reasons should be informed about its potential risks and discouraged. The newborn should be provided with comfort therapies except in those extremely exceptional cases in which significant vital capacities are shown.

▪ 23 weeks' gestational age (161 - 167 days). Caesarean section based on foetal indication is not recommended. At birth the newborn's vitality must be carefully assessed. Resuscitation must be performed; the decision must be shared with the parents if the newborn shows capacity to survive. When the newborn shows highly compromised clinical conditions the physician will have to take into consideration the advisability of not starting or continuing extraordinary therapies, which would be out of proportion to the objective of the best interest of the little patient. Obviously this decision will also have to be shared and assessed with the parents. Ordinary therapies must always be provided to these infants, namely comfort assistance.

▪ 24 weeks' gestational age. Caesarean section may be exceptionally taken into consideration for foetal reasons. Intensive treatment of the newborn is more advisable than at 23 weeks, but always on the basis of favourable objective clinical criteria which suggest proceeding with extraordinary therapies, such as the presence of attempts at respiration, valid cardiac frequency, recovery of skin colour.

▪ 25 weeks' gestational age. Caesarean section may be performed for foetal reasons. Newborns must be resuscitated and subjected to intensive, extraordinary therapies, except those cases presenting severely compromised clinical conditions suggesting the impossibility of survival.

An intense debate arose in response to the *Carta di Firenze *[10,11] with the National Bioethics Committee (NBC) influentially taking sides, considering "ethically" and scientifically unacceptable the presumption of identifying a temporal threshold below which to refuse, a priori, any attempt at resuscitation. The NBC, however, recognise in the chart itself the merit of drawing attention to the problems of neonatology and of insisting on the importance of palliative therapies for extremely premature newborns and on their right to serious antalgic therapies and to a death with dignity. In the *Carta di Firenze *the time of intrauterine development is referred to as the most indicative parameter of maturation, that is to say of potential vitality, without taking into account the conditions which have caused such a premature birth (spontaneous interruption of pregnancy due to accidental or pathological causes; spontaneous or induced multiple pregnancy; uterine malformations; foetal malformations; etc.). The chart explicitly chooses to make reference to extremely low gestational age newborns (22-25 weeks) for whom it proposes the "do not resuscitate order" as a rule of desirable behaviour at 22 weeks and below, whilst allowing for departures in exceptional cases (such as the presence of spontaneous respiratory acts, efficient heartbeat, recovery of skin colour). Moreover, it considers gestational weeks of 23-24 weeks as a grey zone of uncertain vitality. The NBC, whilst acknowledging the exactness of the scientific premise of the extremely low survival rate at ≤ 22 weeks, admonishes that this may lead to behaviours prejudicially "not resuscitating". The critical point of the *Carta di Firenze*, according to experts at the NBC, is the difficulty of establishing truly reliable parameters which would provide the certainty of prognosis at birth. Therefore, the assessment at birth of vital parameters can not have a rigorous prognostic value and cannot justify an aprioristic decision to desist from therapy. One can and must have doubts, obviously of a merely probabilistic character, about diagnosis and prognosis made in the first hours of life. Finally, it is the opinion of the NBC that the possibility of the newborn's life, once resuscitated, continuing with a handicap of major or minor severity, does not mean the treatment performed is futile. In other words, a treatment which prolongs the life of a disabled person cannot be defined futile simply because it prolongs a life considered by some to be of low quality. On the opposite side many Authors believe that the future quality of life of a newborn must be taken into account in deciding the best treatment for a very ill neonate [12,13].

Moreover, the NBC invites us to consider the modalities of treatment of extremely premature newborns in a way absolutely analogous to the assessment of any other form of treatment to which handicapped people are subjected, regardless of their age. Finally the NBC refers to the Italian legal system. Law 194/1978, which allows the voluntary interruption of pregnancy, provides that, when there is the possibility of autonomous life of the foetus, regardless of gestational age, the physician who has performed the intervention must put into effect all the appropriate procedures to guarantee survival, in compliance with the principle of equality. Like any other person needing assistance, extremely premature newborns have full right to the adoption of all the appropriate procedures to ensure their survival. The Carta di Firenze instead seems to invert this principle for newborns between 22 and 23 weeks, who appear to deserve resuscitation practices only exceptionally, when there is evidence of significant vital capacities or of the capacity to survive. This inversion does not appear ethically acceptable to the NBC. Uncertainty about the prognosis of these newborns seems to give rise to an inversion of the general rule: not the duty to assist as a general rule anymore, except when there is evidence of the futility of the intervention given the incapacity of the newborn to live autonomously outside the mother's body, but the opposite prescription, according to which lifesaving assistance would not be due in general, but only in exceptional cases where the newborn shows significant vital capacities or evidence, at the 23rd week, of capacity to survive.

According to the chart it seems that this requirement should be accompanied by the parents' consent. Starting from these assumptions, the NBC believes that with birth every newborn, albeit extremely premature, acquires the juridical status of a person and consequently has the full right to therapies. Therefore it is to be hoped that in general criteria to be adopted for resuscitation of newborns is no different from those adopted to resuscitate an infant no longer in the neonatal phase, or an adult.

The prognostic uncertainty of the time between 22 and 23 weeks of gestation cannot justify a rigid assumption of the futility of resuscitation and the impossibility of demanding the physician's duty to adopt every appropriate measure to protect the newborn's life. The physician may well abstain from this duty but only by considering each individual case and diagnosing the insufficient vitality of the newborn. The NBC believes that an extremely premature newborn should not be resuscitated when this practice objectively assumes the tones of therapeutic obstinacy, even if the prolongation of therapeutic interventions is strongly urged by the parents.

Finally, a point of fundamental importance is the centrality of parents in the decision-making process regarding the therapies administered to their premature newborns. When parents disagree with the physician's assessments favourable to resuscitating the newborn it is the opinion of the NBC that the physician's opinion must prevail.

Other documents have been issued in the same period in Italy. We refer in particular to the so-called "*Carta di Roma*", drawn-up in February 2008 and signed by the Directors of the Obstetric and Gynaecologic Clinics and by numerous neonatologists from the four Medical Faculties of the Universities of Rome. This document suggests to "treating extremely premature newborns as any person in a condition of risk and assisting them in an adequate way", regardless of their gestational age, thus suggesting an approach which is not based on a statistical criterion, like the rate of survival or disability, but which must be individualized. Almost contemporaneously, the Italian Superior Council of Health (March 2008) expressed its opinion "regarding the advisability of identifying protocols for perinatal therapies in extremely low gestational ages, to define the temporal ranges and modalities of assistance most appropriate to guarantee the safeguarding of the health and dignity of the newborn and its mother, in accordance with more recent scientific evidence". In the final recommendations we read that "taking into account that medical and resuscitative treatment can not be confined in rigid schemes, but requires a careful and individualized assessment of clinical conditions at birth ... after the assessment of their clinical conditions ... the appropriate resuscitation procedures [must be] guaranteed to newborns, with the aim of revealing potential vital capacities, which can predict the possibilities of survival following intensive care". If clinical evolution were to show that the intervention is ineffective, intensive therapies turning to therapeutic obstinacy should be avoided. In any case newborns would be provided with nutrition and hydration compatible with their clinical conditions and with other compassionate therapies, always treating them with an attitude of respect, love and care. The therapies administered to the newborns should always respect the natural person's dignity, ensuring the most appropriate interventions to safeguard their potential development and the best quality of life possible. It being understood that resuscitation treatment requires immediate decisions and prompt and timely actions, the parents should be provided with understandable and exhaustive information about the newborns' conditions and their life expectancy, offering them understanding and all possible psychological support. In the event of a conflict between the parents' requests and the physician's decision, a shared solution should be sought, taking into consideration the safeguard of the foetus and newborn's life and health.

The Italian Society of Gynaecology and Obstetrics [14] underlined that at birth it is impossible to define a sure prognosis only on the basis of the newborn's gestational age and weight, even taking into account the possibility of error in the assessment of gestational age and that at low gestational ages the possibilities of survival increase very quickly as days go on. Thus, besides gestational age and weight, it is necessary to consider every single case on the basis of the newborn's vitality, of his/her reactions to tactile and environmental stimulation and of his/her development. Moreover, it is recommended as necessary to agree on medical intervention with the parents, having provided them with adequate information; neonatal resuscitation, pre-alerted and adequately shared by a multidisciplinary team (obstetrician, neonatologist and anaesthetist), offers the newborn increasingly improved resources.

Finally, in the scenery that animates the current Italian debate on these matters, the stance sustained with particular emphasis by the exponents of the Catholic ethics appears strong. This assumes that the idea of the "holiness" of human life is fundamental and has expressed clear positions regarding the *Carta di Firenze*, emphasising the fact that in this document the right to life gives way to its "quality". To an increase in births of premature infants corresponds a constant tendency to not resuscitate the most severe cases among them, despite the fact that they have the possibility to survive. Against this tendency, it is sustained an approach based on respecting the person and on the adequacy of the means to employ in order to avoid any form of therapeutic obstinacy which in any way can not include the assessment of the quality of the newborn's future life. Many voices raise in the Italian panorama to underline how the abstention from neonatal resuscitation is an alarming phenomenon because it ends up with depriving of a chance someone who might have it. Without doubt, the importance attributed by a physician to religion in his or her life was also consistently associated with decision making: being Protestant or having no religious background is consistent with a lower tendency to actively resuscitate premature neonates [15].

## Discussion

From this scenario that reflects the current animated debate in Italy, derives the intrinsic difficulty of laying down any rigid guidelines on the subject, especially when gestational age is taken as a reference parameter for medical decisions. First, there is a fair margin of error in the estimation of gestational age, so that in dubious cases, the *Carta di Firenz*e for example specifies the "fundamental importance of clinical evaluation of the newborn by a neonatologist, who should take into account particularly the newborn's conditions at birth, obstetric history and response to resuscitation procedures". Recent studies [16] based on data of pregnancies induced with IVF programmes (the only case in which dating can be absolutely sure), show that the best echographic performance in the dating of pregnancy may show an error oscillating between -10 and +7 days. The timing of the menstrual cycle is, obviously, even more inaccurate, reaching deviations of as much as 14 days [17]. There are obvious repercussions of such a range of error on the subtle decisional distinction right in that grey zone in which even a temporal oscillation of a few days may lead to abstention from neonatal resuscitation procedures or, conversely, the initiation of resuscitation therapies. On the other hand, neonatal prognosis is conditioned by many independent predictive factors (birth weight, use of steroids before birth, multiplicity of pregnancy) [18]. It is therefore evident that the chronological criterion of gestational age alone might lead to a dangerous simplification in evaluating decisional paths, giving an excessive value to one single parameter. It is well known that the prognosis for very preterm children varies with the place of birth (level III perinatal center or not), the attitude of both obstetricians and pediatricians toward care and hence the interventions they use, gestational age, postnatal age, and then later comorbidities [18]. On the other hand, many Authors still consider gestational age, although imperfect, the best parameter, indicator of the infant maturation (that means of the survival capacity) and all the existing Guidelines refer to gestational age to recommend behaviours and clinical choices [19,20].

In the bioethical debate it was underlined how one of the unique features of neonatal bioethics is its focus on guidelines that specify which extremely preterm babies should not receive resuscitation [21]. No other area of medicine has been as focused upon such policies or as specific in delineating treatment limitations. Instead, in other areas, guidelines are broad and general, with much room for individual clinical judgement and professional discretion. What some authors find, in fact, is that policies for newborns appear very different from those for other patients populations [17]. In fact, even in critical situations burdened by high mortality or morbidity (for example adult patients with cardiac arrest after trauma; cardiac arrest in children following severe trauma; adult patients with a primary haemorrhagic stroke) [22], the low percentages of survival or even the prevision of long term significant and disabling sequelae certainly do not lead to abstention from the resuscitation procedures laid down in rigorous protocols or guidelines [23,24].

## Summary

The crux of the matter is whether strict guidelines, reference standards based on the gestational age parameter, and authority rules are necessary [25,26]. We believe that the right answer to this question is "no". In a decisional sphere burdened by such limited prognostic certainties, an individual approach is infinitely more acceptable than a statistical approach: any decision ought to be based upon the individual circumstances of each newborn rather than on reference to guidelines, especially if these are based on gestational age. Physicians do not need rigid rules based on inflexible gestational age and birthweight guidelines but guidance to address the difficult and trying issues associated with infants born at the margins of viability with a realistic assessment of the infant's clinical condition [27].

We firmly think that as the principles regarding treatment of low or very low gestational age newborn should be the same of those followed for other patients, there is no need for specific policy statement. Generally, the purposes of guidelines have to improve knowledge regarding neonatal outcomes, to provide consistency in periviability counseling, and to promote informed, supportive, responsible choices. Flexible guidelines are well accepted and can be used by all neonatologists, obstetricians, and nurses who provide care to pregnant women and infants at extremely early gestational ages but resuscitation decisions for estremely preterm infants should be approached in the same way as for other patients. They should be individualized with objective and the most accurate individual prognostication, taking into account all the relevant clinical characteristics [17].

In conclusion, the physician has the responsibility to make an assessment of the infant's condition at birth and of the baby's response to the clinical intervention provided, and then a judgement on whether or not to initiate resuscitation. All subsequent decisions are to be jointly made on the basis of the infant's best interests, with clinicians and parents entering into what has been described as a "partnership of care" [28,29].

## Competing interests

The authors declare that they have no competing interests.

## Authors' contributions

Both the Authors have developed the argument, reviewed the literature and prepared the article's manuscript for submission.

## Authors' informations

**Professor Emanuela Turillazzi MD, PhD **is Associate Professor of Legal Medicine at the University of Foggia, in Italy. Her main research interests are in Medical Ethics, Forensic Pathology, and Medical Responsibility. She is author and co-author of a large number of papers published in major scientific journals and of the book *Medical Responsibility and Procreation Techniques *published in 2007 by Giuffrè Editore, Milan, Italy.

**Professor Vittorio Fineschi MD, PhD **is Full Professor of Legal Medicine at the University of Foggia, School of Medicine, Italy. In 1988, 1989, and 1993 he won the National Awards for the best scientific paper of the Year from the Italian Society of Legal Medicine. His main research interests are generally in Medical Ethics and Forensic Pathology. His personal impact factor is 150,648 (ISI 2008).

## Pre-publication history

The pre-publication history for this paper can be accessed here:

http://www.biomedcentral.com/1472-6939/10/19/prepub
